# Role of Apoptosis in disease

**DOI:** 10.18632/aging.100459

**Published:** 2012-05-31

**Authors:** B. Favaloro, N. Allocati, V. Graziano, C. Di Ilio, V. De Laurenzi

**Affiliations:** ^1^ Dipartimento di Scienze Biomediche, Universita' “G. d'Annunzio” Chieti-Pescara, 66100, Chieti, Italy; ^2^ Fondazione “G. d'Annunzio”, Centro Studi sull'Invecchiamento, Ce.S.I., 66100, Chieti, Italy; ^3^ BIOUNIVERSA srl, University of Salerno, Fisciano (SA), Italy

**Keywords:** cell death, caspases, apoptosis, cancer, aging, diseases

## Abstract

Since the initial description of apoptosis, a number of different forms of cell death have been described. In this review we will focus on classic caspase-dependent apoptosis and its variations that contribute to diseases. Over fifty years of research have clarified molecular mechanisms involved in apoptotic signaling as well and shown that alterations of these pathways lead to human diseases. Indeed both reduced and increased apoptosis can result in pathology. More recently these findings have led to the development of therapeutic approaches based on regulation of apoptosis, some of which are in clinical trials or have entered medical practice.

## INTRODUCTION

Since its initial description cell death has appeared as a basic biological phenomenon fundamental for development and regulation of tissue homeostasis whose alteration has important implications in pathology [1]. Indeed cell death contributes to tissue homeostasis by balancing mitosis and pathology can derive from both its increase or decrease. Since the initial description of cell death in the 1960s a number of different death mechanisms have been described and have been classified both on morphological and biochemical criteria. A recent paper published in CDD [2] by a large number of experts in the field has suggested a classification of the different types and is a good reference for the subject. This review will mostly focus on the role in different human diseases of caspase dependent apoptosis Table 1).

**Table 1:** Diseases in which alterations of apoptosis are involved

**Cancer**	
Breast	[20, 28, 160, 161]
Lung	[21, 34, 162]
Kidney	[22]
Ovary and uterus	[28]
CNS	[13, 23, 25, 163-165]
Gastro-enteric trait	[24, 30-32, 166]
Head and Neck	[167]
Melanoma	[33, 35, 168-170]
Lymphomas	[18, 19]
Leukemia	[26, 171-178]
**Neurological disorders**	
Alzheimer	[55, 179-183]
Parkinson	[63, 184, 185]
Huntington	[186-188]
Amyotrophic Lateral Sclerosis	[189-191]
Stroke	[192-194]
**Cardiovascular disorders**	
Ischemia	[99, 195-198]
Heart Failure	[99, 199]
Infectious diseases	
Bacterial	[200-206]
Viral	[150, 207-214]
**Autoimmune diseases**	
Systemic Lupus erythematosus	[153, 215, 216]
Autoimmune lymphoproliferative syndrome	[154, 155]
Rheumatoid arthritis	[217]
Thyroiditis	[1, 217, 218]

**Figure 1:**
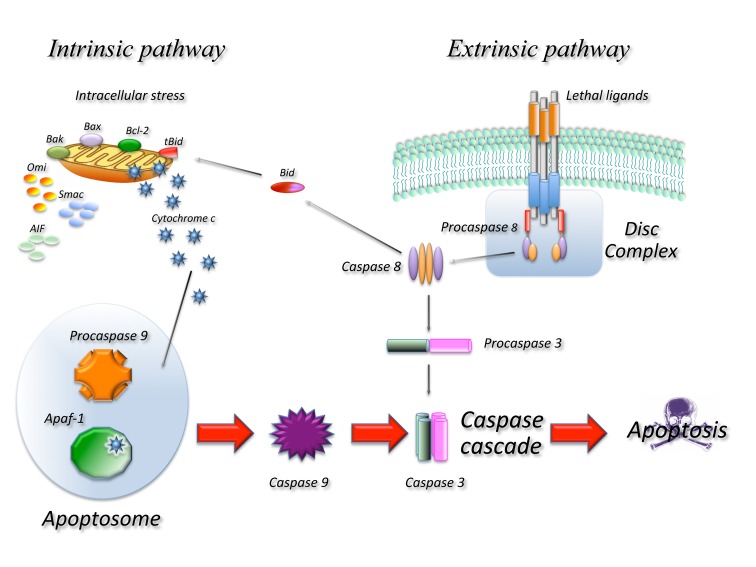
Schematic representation of the main molecular pathways leading to apoptosis In the extrinsic pathway upon ligand binding to specific receptors the DISC complex is formed and caspase 8 activated. In the intrinsic pathway release of cyt c from the mitochondria result in the formation of the apoptosome and activation of caspase 9. Caspase 8 and 9 then activate downstream caspases such as caspase 3 resulting in cell death. The two pathways are connected through the cleavage of the BH3 only protein BID.

Caspase dependent apoptosis (Figure 1) is characterized by the activation of pathways leading to the activation of a family of proteases: caspases resulting in an ordered disruption of the cell without leakage of cellular components and induction of inflammation. Apoptosis occurs following the activation of specific pathways that result in a series of well defined morphological events. The dying cell initially shows nuclear and cytoplasmic condensation, followed by blebbing of the plasma membrane that results in release of small membrane-enclosed particles containing cellular components known as apoptotic bodies. These are rapidly identified by neighbouring cells or professional phagocytes and disposed generally without induction of inflammation or tissue scarring [3]. Apoptosis depends on activation of caspases that will then cleave a number of substrates [4] resulting in the biochemical and morphological changes typical of this form of death. All caspases are synthesized as inactive zymogens that need activation to exert their function. Full activation is achieved through cleavage of a pro-domain generally by other caspases. From a functional point of view we can distinguish two types of caspases: up-stream and down-stream caspases. Up–stream caspases are activated when more enzyme molecules are brought in close proximity and undergo conformational changes upon binding to activation complexes, this results in their cleavage and full activation [5, 6]. Once activated they will activate additional molecules of the same enzyme as well as down stream caspases. Down stream caspases on the other hand can only be activated by cleavage of the pro-domain by up-stream caspase. Two main molecular pathways lead to caspase activation and therefore to apoptosis the so-called extrinsic and intrinsic pathway.

Extrinsic apoptosis indicates a form of death induced by extracellular signals that result in the binding of ligands to specific trans-membrane receptors, collectively known as death receptors (DR) belonging to the TNF/NGF family. All death receptors function in a similar way: upon ligand binding several receptor molecules are brought together and undergo conformational changes allowing the assembly of a large multi-protein complex known as Death Initiation Signalling Complex (DISC) that leads to activation of the caspase cascade. In the FAS/CD95 signalling complex, that can be used as a prototype of this form of death, upon ligand binding FAS recruits, through a highly conserved 80 amino acid domain, known as death domain (DD), an adaptor molecule: Fas-associated protein with a DD (FADD). FADD contains another conserved protein interaction domain known as Death Effector Domain (DED) that binds to an homologous domain in caspase 8 leading to its activation. Active caspase 8 will activate additional caspase 8 molecules as well as downstream caspases such as caspase 3 [7].

The intrinsic pathway is activated in response to a number of stressing conditions including DNA damage, oxidative stress and many others. In all cases this multiple forms of stress converge on the mitochondria and determine mitochondrial outer membrane permeabilization (MOMP) this in turn results in dissipation of the mitochondrial membrane potential and therefore in cessation of ATP production as well as release of a number of proteins that contribute to caspase activation. At least two molecular mechanisms (not mutually exclusive) have been proposed to explain how different signals converge at the mitochondria resulting in MOMP. One involves the pore forming ability of some of the BCL-2 family proteins in the outer mitochondrial membrane [8] and the other is the result of the opening in the inner membrane of the permeability transition pore complex (PTPC), that would require the Adenine Nucleotide Transporter (ANT) and the Voltage Dependent Anion Channel (VDAC) [9, 10]. The Bcl-2 family proteins are essential regulators of this type of apoptosis and are all characterized by the presence of at least one Bcl-2 Homology (BH) domain. From a functional point of view they can be classified in anti-apoptotic members containing three or four BH domains (such as Bcl-2, Bcl-xl, Bcl-w, Mcl-1) and pro-apoptotic members with two or three BH domains (such as Bax, Bak, Bcl-xs, Bok) or with just one (such as Bad, Bik, Bid, Bim, Noxa, Puma). Pro-apoptotic members of the family mediate apoptosis by disrupting membrane integrity either directly forming pores or by binding to mitochondrial channel proteins such as VDAC or ANT, while anti-apoptotic members would prevent apoptosis by interfering with pro-apoptotic member aggregation. The different apoptotic signals are sensed by BH3 only proteins that are induced or activated and migrate to the mitochondria where they bind the pro-survival members of the family removing their block or to the pro-apoptotic members promoting their aggregation [11].

In any case once MOMP occurs a number of proteins are released from the mitochondria, these include Cytochrome C (CYTC), apoptosis-inducing factor (AIF), endonuclease G (endo G), Direct IAP-binding protein with low PI (DIABLO, also known as SMAC) and others. Once CYTC is released it binds to APAF-1 inducing the formation of a large complex, known as the apoptosome, that recruits caspase 9. In the apoptosome, caspase 9 is activated and cleaved and will activate additional molecules of caspase 9 as well as down-stream caspases such as caspase 3. Due to its lethality the system is subject to a number of controls as an example the cytoplasm contains a class of proteins known as Inhibitors of Apoptosis IAPS that bind and inactivate caspases. Upon MOMP the mitochondria also releases proteins such as DIABLO/SMAC that bind to IAPS removing their inhibition and allowing apoptosis to occur.

The intrinsic and extrinsic pathways are not completely independent: in some cells in fact activation of caspase 8 results in activation of the mitochondrial pathway. In this case caspase 8 among other things cleaves a BH3 only protein BID generating a truncated fragment known as truncated BID (tBID) that can permeabilize the mitochondrion resulting in MOMP [12].

### Cancer

Based on its role in maintaining tissue homeostasis it is not surprising that alterations of apoptosis play an important role in cancer development. Moreover defects in the apoptotic pathways are responsible for resistance to therapy and new therapeutic approaches attempting to re-activate these pathways bypassing the block are currently being studied [13-17]. Alteration of many proteins involved in both intrinsic and extrinsic signaling pathways have been described and are in part summarized below. It is clear that alterations of up-stream regulators of these pathways are the most common alterations found in cancer cells, as an example p53 that can induce cell death in response to a number of different stress stimuli, is the most frequently mutated gene in human cancers. A description of these defects however is beyond the scope of this review.

#### BCL-2 Family proteins alterations

Bcl-2 was initially isolated and characterized in a subset of B-cell lymphomas. These tumours carry a typical translocation t(14;18) that involves Bcl-2 gene and results in its over-expression. Thus it appeared that this gene played a role in cancer even before his function in regulating apoptosis was clearly defined. Since those early days a bulk of data have proven the role of Bcl-2 alterations in development of cancer including a number of animal models. In fact transgenic mice carrying bcl-2 overexpression are actually susceptible to develop different forms of lymphoproliferative disorders [18].

Bcl-2 has been found overexpressed not only in B-cell lymphomas, but also in a variety of other cancers such as: Hodgkin lymphoma where it seems to be associated with worse overall survival [19]; breast cancer where it correlates with tumor aggressiveness, reduced survival and resistance to endocrine therapy [20]; non-small and small cell lung carcinoma (moreover squamous) [21], renal cell carcinoma [22]. A novel polymorphism of the BCL-2 promoter (-938C>A) associates with increased aggressiveness and worse prognosis in glioblastoma multiform [23], chronic lymphocytic leukemia, as well as with a better survival and outcome in breast and ovarian cancers.

In the last years a variety of alterations of the different Bcl-2 family members have been described and have proven the importance of this group of proteins in cancer development. Bax and Bak mutations [24] have been described in colon and gastric carcinomas. Unexpectedly however neither BAX single KO or BAX/BAK double KO show increased tumour formation suggesting that compensatory mechanisms can allow apoptosis in these cells. Interestingly however Bax/pARF double KO mice exhibit an increased variety of tumours (sarcomas and carcinomas). Furthermore Bax deletion accelerates growth of brain tumours in a p53 mutant mice model [25], and of mammary tumours in a SV40 large T antigen transgenic mouse model. Various BH3 protein alterations have also been implicated in cancer development, as an example Bid-deficient mice are prone to develop a form of chronic myelomonocytic leukemia [26], as well as diffuse large B-cell lymphoma.

The possibility to target Bcl-2 family members proteins to induce apoptosis in cancer cells has been studied for many years now and particular attention has been given to BH3 only proteins in designing drugs that would mimic their pro-apoptotic functions. Some of these are currently being tested in phase I/II clinical trials [27-29]. Antisense oligonucleotides targeting Bcl-2 have also been developed and in one case have reached a phase III clinical trial in patients with chronic lymphocytic leukemia.

#### Apoptosome defects

Apaf1 inactivation can substitute for p53 defects in promoting transformation of myc-expressing cells, moreover it is frequently silenced or inactivated in human cancers. Indeed melanomas, leukemias, glioblastomas, and cervical carcinomas have been shown to down-regulate Apaf1 by epigenetic mechanisms. In addition in some cancers a defective Cyt-C dependent caspase 9 activation in the presence of normal or elevated Apaf1 levels has been reported but the underlying molecular mechanism is still elusive and the existence of an unidentified apoptosome inhibitor has been suggested. Sequestration of Apaf1 into lipid rafts has also been demonstrated in Burkitt lymphoma cells providing another molecular mechanism for apoptosome dysfunction.

#### Death receptor pathway defects

Death receptor pathways alterations have profound implications in cancer and in particular in the mechanism leading to the ability of tumours to avoid immune response. In a simplistic way one can imagine a scenario in which reduced expression of death receptors protects cells from the immune system and increased surface (or soluble form) expression of death ligands allows killing of reactive cells. Indeed CD95 null mice are prone to accumulate abnormal T-cells, with splenomegaly and lymphoadenopathy and CD95 is lost or mutated in several human cancers. In fact CD95 is lost in hepatocarcinomas [30], present in less than 5% of invasive esophageal cancer cells in 79% of patients [31] where it correlates with depth of invasion and nodes metastasis, mutated in adult T cell leukemia, down-regulated in colon cancer [32], ovarian, cervical and endometrial cancers, melanoma (where lymphocyte infiltration of the invasive layer correlates with prognosis) [33] and in more than 90% of lung cancers [34]. In support of the above mentioned model, down-regulation of Fas-L leads to decreased tumor volume and increased lymphocyte infiltration. Over-expression of FAS-L has been reported in hepatocarcinomas, esophageal cancers [31], breast cancers, melanomas [35], astrocytomas , metastatic colon cancers, gastric cancers and in more than 60% of sarcomas (reaching 95% in metastatic ones). The soluble form of Fas-L (sFas-L), was also found in peripheral blood of cancer patients, where it possibly exerts an immunosuppressive effect [36, 37].

Defects of the CD95 signaling pathway can also be a consequence of alterations of other components of the DISC. FADD mutations were reported in non-small-cell lung cancer [38] and complete loss was described in a subset of diffuse large B-cell lymphoma. However FADD defects have also been shown to have a pro-oncogenic function, probably due to a role of this protein different from apoptosis. As an example phosphorylated-FADD in lung adenocarcinomas correlates with poor survival [39], and its overexpression associates with poor prognosis in oral squamous cell carcinomas.

A number of evidence highlight the importance of the Trail receptor pathway in tumour onset and development. The importance of Trail as a tumour suppressor is supported by a number of results in different animal models including the onset of spontaneous haematopoietic malignancies in Trail KO mice [40]. As described for CD95 also in the case of Trail, defects mostly allow tumour cells to escape immune control. Trail receptors 1 and 2 map to chromosome 8p21-22 frequently lost in tumours and mutations of trail receptors have been described in up to 20% of various human tumours, including breast cancer, head and neck cancers, and non-Hodgkin lymphomas.

The possibility to develop cancer therapies based on the activation of death receptors has been attractive since their discovery, however toxicity of therapies targeting TNF and CD95 have greatly reduced the initial enthusiasm. The finding that recombinant Trail preferentially induced apoptosis in cancer cells while sparing normal cells supported by its low or absent toxicity in vivo [40, 41] have attracted growing interest on the possibility to exploit this pathway for cancer therapy. Despite a large investigative effort however the reason for this selective activity is poorly understood.

#### Altered caspase activity

Caspases are the final effectors of both extrinsic and intrinsic apoptosis, therefore it is expected that interfering with their function impairs these pathway leading to a survival advantage for cancer cells and indeed caspase alterations are not rare in a variety of tumours. These can be due to mutations, promoter methylation, alternative splicing, and post-translational modifications. Some of these defects are loss of functions, but in other cases mutated caspases act as dominant negatives preventing activation also of the wild type protein [42].

Altered caspase function can also be a consequence of modified expression of their specific inhibitors, as an example cFLIPs that competes with caspase 8 for FADD binding, thus preventing its activation, is often elevated in tumours, while its down-regulation can sensitize tumour cells to therapy. Among caspase inhibitors an important role is played by IAPs. Indeed alterations of IAPs are found in a variety of human cancers and are associated with poor prognosis and resistance to therapy. In some cases however loss of IAPs correlates with tumour progression complicating the issue and suggesting that the role of IAPs has to be carefully evaluated based on cell context. While initially described as caspase inhibitors now IAPs have been recognized to regulate a multitude of other cellular functions including regulation of the immune response cell migration, mitosis and proliferation [43]. As many of these processes are often modified in cancer it is clear how alteration of IAPs can play a role in tumorigenesis not only as a consequence of altered apoptosis. In fact probably the most important pathway regulated by IAPs that contributes to cancer development is the NF-kB signaling pathway. XIAP, cIAP1 and cIAP2 have been shown to regulate this pathway and as a consequence inflammation, immunity and cell survival. Moreover cIAPs protect from TNF killing. In addition, recent findings show a role for IAPs in metastatization as a XIAP/survivin complex would trigger NF-kB pathway leading to activation of cell motility kinases [43]. This however is still a controversial issue and other studies show a suppressive effect of IAPs on cell mobility.

In any case due to their involvement in cancer progression and to their ability to suppress apoptosis IAPs have become an attractive therapeutical target, leading to the development of IAP inhibitors, some of which are based on natural inhibitors such as Smac/DIABLO [43, 44]. These drugs appear to be able to directly kill cancer cells or at least sensitize them to other killing agents while sparing normal cells. A number of these compounds are currently entering clinical trials and their efficacy will be evaluated in the next few years.

### Neurological disorders

From a physiological point of view apoptosis plays a key role in central nervous development [45, 46], while in adult brain it is involved in the pathogenesis of a number of diseases including neurodegenerative diseases and acute injury (i.e. stroke).

### Neurodegenerative diseases

***Alzheimer's disease (AD)*** is the seventh leading cause of all deaths in the United States, it is a progressive neurodegenerative disorder characterized by accumulation of amyloid-β β peptides in extracellular senile plaques intra-cellular neurofibrillary tangles (NFTs) formation resulting from hyper-phosphorylated microtubule-associated protein tau resulting in loss of neurons and consequent progressive dementia [47-49].

Neuronal apoptosis plays an important role in AD pathogenesis and caspases seem to be involved also in some of the upstream pathological events. Exposure of cultured hippocampal neurons to β results in caspase 3 activation and apoptosis [50]. Aβ is generated following sequential cleavage of the amyloid precursor protein (APP), and caspase 3 is considered the predominant caspase involved in APP cleavage [51, 52]. Tau protein is also a substrate for caspase 3; cleavage of tau at its C-terminus would promote tau hyper-phosphorylation and accumulation of NFTs. Moreover, β-induced caspase 3 activation causes abnormal processing of the tau protein in models of AD [53]. APP is also cleaved by caspase 6 in vivo [54], moreover the N-terminal APP fragment is a ligand for death receptor 6 (DR6 also known as TNFRSF21) activation of which triggers caspase 6 dependent axonal degeneration [55].

The potential benefit of inhibiting the intrinsic apoptotic pathway has been suggested through the use of a triple transgenic AD mouse model wherein overexpression of the anti-apoptotic Bcl-2 gene blocked activation of caspases 9 and 3; in these conditions, the degree of caspase cleavage of tau was limited, the formation of plaques and tangles was inhibited, and memory retention was improved [56, 57].

***Parkinson's disease (PD)*** is considered the 2nd most common chronic neurodegenerative disorder after AD, it is associated with movement disorders, tremors, and rigidity and is characterized by a specific loss of dopaminergic neurons of the substantia nigra. This degeneration leads to the formation of fibrillar cytoplasmic inclusions known as Lewy bodies. A preponderant role of the aberrant activation of intrinsic and extrinsic apoptotic pathways in PD pathogenesis has been suggested. The involvement of caspases 1 and 3 in apoptotic cell death has been proved using PD animal models [58]. PD has been linked to mutations in several genes such as parkin [59], DJ-1, and a gene codifying for a mitochondrial kinase, (PTEN)-induced kinase 1 (PINK1) [60]. PINK1 function is related to the inhibition of mitochondria-dependent apoptosis [61]. In human and mouse neurons deleted for PINK1 Bax translocation to the mitochondria and cytochrome c release to the cytoplasm occur earlier than in control cells. Furthermore loss of PINK1 results in elevated levels of caspase activation (caspases 3 and 9) [61]. Gene-expression profiling studies performed on material from patients affected by PD confirmed down-regulation of PINK1 as well as other anti-apoptotic proteins such as Bcl-2 but also found evidence for the involvement of the extrinsic pathway. Indeed death receptors such as FAS, TNFRSF10B and TNFRSF21 were up-regulated in PD-affected neurons [62, 63].

***Huntington's disease (HD)*** is a disorder characterized by a degenerative process, which affects medium spiny striatal and cortical neurons. HD is an autosomal dominant disease caused by a mutation in the gene encoding the huntingtin protein (htt); this mutation is responsible for abnormal expansion of a trinucleotide CAG repeat encoding polyglutamine tract expansion in the N terminus of htt [64]. The expanded polyglutamine alters protein folding, leading to generation of aggregates in neurons that seem to be crucial for the neurodegenerative process [65, 66]. Mutant htt is cleaved by different proteases including caspases [67] and accumulation of caspase cleaved fragments is an early pathological finding in brains of HD patients [68]. Moreover transgenic mice models have demonstrated that caspase 6 cleavage of mutant htt is required for the development of the characteristic behavioral and neuro-pathological symptoms. In addition activation of caspase 6, is observed before the onset of motor abnormalities in HD brains, suggesting that these activation could be used as an early marker of the disease [69].

An additional molecular mechanism involves htt-interacting protein 1 (HIP-1) that binds a polypeptide named Hippi (HIP-1 protein interactor) forming a complex that can activate caspase 8. The free cellular HIP-1 concentration is increased when htt is mutated (HD), this would favor the pro-apoptotic Hippi-Hip complex formation [70, 71].

***Amyotrophic lateral sclerosis (ALS)*** is a progressive neurodegenerative disease characterized by muscle atrophy, paralysis, and, death due to progressive loss of motor neurons [72]. About 10% of cases are familial as a result of mutations in the copper-zinc superoxide dismutase (SOD1) gene [73], whereas the majority of them are sporadic. SOD1 catalyzes conversion of the superoxide anion to hydrogen peroxide, however the mechanism by which SOD1 mutations cause ALS is still not completely understood. Several alterations have been identified in ALS and are thought to play a role in motor neuron death, including: neurofilament abnormalities, aggregate formation, oxidative stress, and inflammatory processes [74]. Mice overexpressing a human mutant SOD1 develop neuron degeneration, and have been used as a model [75, 76]. These mice show increased p38 activity that determines increased NO production that in turn results in increased FasL expression and activation of the extrinsic pathway [77]. In addition mutated SOD would localize to the mitochondria and directly trigger CYTC release and therefore neuronal death [78].

Motor neurons degeneration in ALS is also accompanied by inflammation, but the exact mechanism triggering inflammatory response remains unclear. Meissner et al have recently reported that mutant SOD1 induces IL-1 beta and promotes caspase 1 activation resulting in neuro-inflammation that would contribute to the pathogenesis [79]. Finally caspase 1 would act as a chronic activator of caspase 3 contributing to neuronal loss [80].

### Acute CNS insults

***Stroke*** is the leading cause of acquired adult disability in USA [81]. Ischemic injury is caused by the loss of blood flow to the brain, usually as a consequence of an embolism. The decrease in perfusion determines both apoptotic and necrotic neuronal cell death in the affected region (core) due to energy depletion [82, 83]. Around this area of tissues that is irreversibly lost there is an area of partially damaged tissue known as penumbra that triggers local inflammation. Several evidences suggest that inflammation is a crucial event in the progression of ischemic brain damage. Cyclooxygenase-2 (COX-2) induction and prostaglandin E2 elevation have been reported to occur after cerebral ischemic insult [84]. Takadera et al showed that prostaglandin E2 directly induced apoptosis in hippocampal neurons through the activation of caspase 3 [85], suggesting that a direct effect of prostaglandin E2 on hippocampal neurons was mediated by activation of the EP2 receptors. The deletion of EP3 receptors is known to ameliorate stroke injury in experimental stroke models [86], and recently it has been demonstrated that EP3 receptors are involved in the enhancement of inflammatory and apoptotic response in the ischemic cortex [87].

An important role in ischemic brain damage seems to be played also by activation of the receptor pathway. TNF deletion in mice protects the brain from ischemic damage [88]. Fas and FasL levels seem to be increased during brain ischemia [89], and interfering with the Fas signaling pathway using a blocking anti-FasL antibody markedly reduces death of neurons and improves functional recovery in animal models of stroke and spinal injury. Moreover chronic extrinsic cervical spinal cord compression leads to Fas-mediated apoptosis of neurons [90].

C-Jun N-terminal protein kinase (JNK) signaling pathway is known to be activated in response to stress and ischemia. JNK activation precedes inflammation and apoptosis in neuronal cells [91]. Indeed, JNK is involved in the regulation of several pro-apoptotic proteins such as Bim and inhibition of JNK activity attenuated Bax translocation in ischemic neurons [92].

The involvement of caspases in mediating ischemic neuronal cell death has been also demonstrated using caspase 1 and 3 KO mice. Cortical neurons from caspase 3 -/- mice subjected to oxygen-glucose deprivation, were more resistant to cell death [93], while caspase 1 deletion in mice led to reduced production of IL-1 beta [94]. Consistently the reduction of IL-1 beta function, using specific antagonists, results in neuroprotective effects in stroke animal models [95].

Benchoua et al. proposed activation of different and specific pathways in the core or in the penumbra area of the brain infarction. In particular, after cerebral infarction, in neurons of the core area the first apoptotic events are mediated by ligand binding to specific death receptors leading to caspase 8 activation. In the penumbral area, where mitochondria provide residual energy supply, neuronal death is instead induced through the mitochondrial pathway [96].

The reperfusion of ischemic tissue usually improves clinical outcome of patients, but in others it may amplify brain damage due to the ischemia-reperfusion injury. Reactive oxygen species (ROS) levels are immediately increased after a vessel occlusion is cleared, and are considered the main mediators of reperfusion injury [97] resulting in the release of cytochrome C [98].

### Heart diseases

Adult cardiomyocites are post-mitotic cells, therefore this tissue has limited response capability to damage. In general acute damage results in cell death of various types while chronic stress mainly results in hypertrophy and myocardial remodelling. Increased evidences suggest that slow turnover exists in the normal myocardium sustained by stem cells. In pathological conditions however death exceeds mitosis resulting in heart failure. Apoptosis is very rare in normal myocardium with a reported rate of 0.001-0.002% however it is increased in both acute and chronic heart pathologies were it seems to play an important role [99].

#### Ischemia reperfusion

Occlusion of coronary arteries results in myocardial infarction characterized by massive cell death due to deprivation of oxygen, nutrients and survival factors. Reperfusion of the ischemic tissue is the treatment for acute coronary syndromes and limits the size of the lesion but still results in damage due to oxidative stress, increased cytosolic and mitochondrial calcium levels and inflammation determining what is known as reperfusion injury. Apoptosis plays an essential role in the pathogenesis of I/R and in general prolonged ischemia determines an increase in necrosis while reperfusion leads to increased apoptosis. Death during ischemia/reperfusion (I/R) is of different kinds: necrotic apoptotic and autophagic. Both the intrinsic and extrinsic apoptotic pathway are involved in I/R. Indeed lpr mice (FAS deficient) show reduced infarct size while deletion of both TNFR1 and TNFR2 appears to have a protective effect. The role of the intrinsic pathway is demonstrated by reduced apoptosis in cardiomiocytes over-expressing Bcl-2 or lacking Bax, both in vitro and in vivo. As explained above the two pathways are connected through Bid cleavage that is indeed observed in I/R and Bid KO mice show reduced infarct size following I/R. Similarly deletion of PUMA (a BH3 only protein activated by p53) also protects from apoptosis suggesting the involvement of the p53 pathway [99]. A number of studies have shown that inhibitors of caspases can protect cardiomyocites reducing the infarct size. Interestingly over-expression of constitutively active Akt or pharmacological sustained activation, shows significant protection from apoptosis probably by inactivating a number of pro-apoptotic proteins [100].

Ischemia damages several mitochondrial components including proteins of the oxidative phosphorylation complexes and membranes and predisposes them to ROS generation [101]. Indeed it is well known that the production of ROS is greatly increased during the reperfusion phase when oxygen becomes available and the mitochondrial respiratory chain is impaired. Furthermore this is exacerbated by reduced antioxidant defenses.

#### Chronic Heart Failure

Heart failure is a complex syndrome where the heart is incapable of o meeting the metabolic requirement of the body, it can be the result of a number of other heart diseases such as myocardial infarction, cardio-myopathies and hypertension. In all cases it is characterized by left ventricular remodeling with chamber enlargement and wall thinning. During heart failure cardiomiocytes show a modest increase in apoptosis, that however seems to play an important role in the pathogenesis [99]. The estimated rate of apoptosis in cardiomiocytes in patients with dilated cardiomyopathy is between 0.08% and 0.25% and increasing apoptosis by only 0.023% in cardiomyocites by over-expression of caspase 8 in mice results in dilated cardiomyopathy after 2-6 months, that is reduced by treatment with caspase inhibitors [102]. Multiple stress stimuli have been implicated in activating apoptosis in cardiomyocites eventually leading to heart failure, these include: excessive mechanical stress, ROS, β1 adrenergic receptor agonists, angiotensin II, cytokines. The ASK 1-JNK pathway seems to play an important role in mediating ROS dependent apoptosis in these cells (in part trough inactivation of Bcl-2) and regulating left ventricular remodeling. Indeed it has been proposed that inhibition of Ask-1 can attenuate heart failure [103]. Another important pathway seems to involve Gαq, that transduces the signal of a number of receptors including angiotensin II receptor and α1-adrenergic receptor. Over-expression of Gαq in mice heart results in heart failure, this is accompanied by over expression of Nix/Bnip3L, a BH3 like protein, that can induce cardiomiocyte apoptosis [99].

### Infectious diseases

Pathogenic microorganisms, once inside a host must avoid detection and destruction for as long as possible. Several pathogens are able to trigger or inhibit apoptosis in eukaryotic host cells thus escaping the immune system. On the other hand the host can use apoptosis in an attempt to defend himself from pathogens. Thus, apoptosis has a fundamental role in cellular host-pathogen interactions that depends on the nature of the pathogen, the cell type and the intensity of the infection.

### Bacterial infections

Although it is outside the scope of this review, it is worth mentioning a physiological role for apoptosis in bacteria [104]. While initially counterintuitive, several studies have underlined how this process may be fundamental to bacterial physiology. As an example, autolysis, the most common known form of apoptosis in bacterial cells, is used by Bacillus subtilis during sporulation to eliminate a barrier, the cell wall that could interfere with spore germination. Also, when exposed to harmful conditions as antimicrobial agents, to eliminate defective cells, damaged bacteria self-digest the cell wall by peptidoglycan hydrolases. Moreover, bacterial apoptosis maintains biofilm homeostasis by balancing cell death and viability. The increasing knowledge of ancestral forms of apoptosis in bacteria will possibly shed light on the evolution and molecular mechanisms of apoptosis in eukaryotic cells. More relevantly to the subject of this review it is well known that bacteria can modulate apoptosis in the host favoring their survival and propagation. Several apoptogenic molecules have been identified in bacteria including common structural components as lipopolysaccharide in Gram-negatives [105], lipotechoic acid the major constituent of the cell wall of Gram-positives and lipoarabinomannan in Mycobacteria [106] as well as virulence factors such as exotoxins [107], cytolysins [108] and hemolysins [109]. Through all these different molecules, bacteria are able to trigger both intrinsic and extrinsic apoptotic pathways, here we report some examples in different types of both endo-cellular and extracellular bacteria.

***Mycobacterium tuberculosis*** continues to be a major cause of pulmonary infection in the world. It is an intracellular parasite capable of establishing a long-term infection. It lacks toxins and exerts its virulence through its cell wall components [110]. The initial infection typically occurs in the alveolar spaces of the lung where bacteria are phagocytized by macrophages. In the alveolar macrophages, bacteria can survive and replicate by preventing fusion of phagosomes with lysosomes and thus their cellular lysis [110]. The apoptotic response to intracellular pathogens may favour both host and parasite in several ways. The host responds to the presence of intracellular M. tuberculosis triggering a TNF-α-mediated apoptotic pathway. On the contrary, bacteria block the TNF-α signalling by up-regulating the anti-apoptotic protein Mcl-1 [111]. Recently it has been reported that mycobacteria may trigger a non-classical type of apoptosis to exit host cells [112]. Cell death is induced when a threshold of about 20 bacteria per macrophage is reached and begins with some typical features of apoptosis followed by secondary necrosis and release of bacteria. To indicate this non-classical mode of apoptosis, Lee and colleagues coined the expression “high-MOI apoptosis” [111]. In addition Mycobacteria secrete a 19kDa lipoprotein that binds the TLR2 receptor that signals through myeloid differentiation factor 88 (MyD88) activating apoptosis in macrophages [113].

***Pseudomonas aeruginosa*** is an opportunistic, multidrug resistant bacterium that often causes severe nosocomial infections such as urinary tract infections, pneumonia and bacteremia [114]. P. aeruginosa is also responsible for pulmonary infection of patients with cystic fibrosis [114]. Although considered as an extracellular pathogen, P. aeruginosa can invade and survive within different types of cells, in particular epithelial respiratory cells. P. aeruginosa triggers cell death of lung epithelial cells through the CD95 pathway [115]. In vivo experiments have demonstrated that, in this case, apoptosis plays a role in the host defence against the bacterial infection [115]. Usually, P. aeruginosa is aspired into the lower respiratory tract after colonizing the oral cavity where oral bacteria may facilitate the adhesion and invasion of P. aeruginosa into respiratory epithelial cells followed by cytokine release and apoptosis [116]. Indeed co-incubation with oral bacteria resulted in increased induction of apoptosis in host cells [116].

***Chlamydiae*** are obligate intracellular bacteria that have adapted to endo-cellular life to the extent of no longer being able to replicate outside the host. *Chlamydia trachomatis* is a common sexually transmitted human pathogen that causes urogenital infections, conjunctivitis and trachoma, while *Chlamydophila pneumoniae* is commonly associated with upper respiratory tract infections and can give rise to community-acquired pneumonia [117]. Apoptosis-inhibiting mechanism have been shown in both and appear to be similar [105]. During persistent infection, *Chlamidiae* inhibit the release of cytochrome c thus blocking apoptosis [118]. The underlying molecular mechanism seems to be the proteolytic degradation of BH3-only proteins by a chlamydial protease-like activity factor (CPAF) [119].

***Enteric pathogens producing Shiga toxins*** Shiga toxins (Stxs) consist of a family of related cytotoxic proteins expressed by the enteric pathogen *Shigella dysenteriae* serotype 1 and by several serotypes of *Enterohaemorrhagic Escherichia coli* (EHEC; Shiga toxin-producing E. coli, STEC), *Shigellae* are endocellular human-adapted pathogens transmitted by the fecal-oral route. STEC are frequently associated with severe disease, while not invasive they are able to adhere to the intestinal epithelium, causing severe cell alterations. Stxs inhibit protein synthesis by inactivation of eukaryotic ribosomes [120], they have an AB5 structure consisting of an enzymatically active A-subunit and a pentameric B-subunit that bind the toxin receptor globotriaosylceramide (Gb3), a neutral glycolipid of the globo-series. Stxs are transported in a retrograde manner, through the Golgi, to the endoplasmic reticulum allowing the transfer of the A-subunit into the cytosol where it inhibits protein synthesis [121].

*Shigellae* and EHEC can survive phagocytosis by inducing apoptosis through activation of the MAPK pathway [107, 122]. Moreover through the prolonged activation of the ribotoxic and endoplasmic reticulum stress responses, they may initiate apoptosis with the rapid activation of caspase 8 and activation of both intrinsic and extrinsic pathways [107].

Interestingly it has also been demonstrated that Stxs induce apoptosis in cancer cells and therefore could be used as potential anticancer agents [123]. However, an obstacle is constituted by the possible toxicity since they can also inhibit protein synthesis and trigger apoptosis in normal cells.

***Helicobacter pylori*** is the major cause of gastroduodenal diseases including peptic ulcer disease, gastric lymphoma and gastric adenocarcinoma [124]. Vacuolating cytotoxin (VacA) is one of several virulence factors identified in *H. pylori* [124]. In addition to cell vacuolation, VacA has other effects on different cell types, including membrane channel formation, inhibition of T-cell activation and proliferation as well as apoptosis. It has been documented that VacA induces apoptosis through the mitochondrial-dependent pathway in gastric epithelial cells [125]. The toxin binds to the RPTPβ (receptor-like protein tyrosine phosphatase) receptor and activates the pro-apoptotic Bcl-2 family proteins, Bax and Bak. In addition, VacA causes down-regulation of JAK-STAT3 signaling pathway resulting in reduced expression of the anti-apoptotic Bcl-2 family proteins, Bcl-2 and Bcl-X_L_.

***Staphylococcus aureus*** is an ubiquitous bacterium and is a part of the human skin and mucosal flora, it can engender a variety of diseases and it is one of the most common causes of hospital infections. It secretes several virulence factors, exotoxins and enzymes, involved in the pathogenic mechanisms of the bacterium. Two virulence factors have mainly been involved in induction of cell death. Superantigen enterotoxin B directly binds to both MCH class II molecules and to the variable regions of the T-cell receptor beta chain (TCRVbeta), cross-linking them in a non-specific way, resulting in polyclonal T-cell activation which can be followed by cell death [126]. Enterotoxin B causes activation-induced cell death of T lymphocytes, a process involving TCR binding, activation, cell expansion, FAS expression and finally PCD. Staphylococcal α-toxin is an hemolysin that causes pore formation in the host cell membranes, and the consequent disruption of the Na^+^/K^+^ balance appears to trigger apoptosis through regulation of Bcl-2 and CYTC release.

***Streptococcus pneumoniae*** is part of the human respiratory tract normal flora and is the common cause of community-acquired pneumonia, bacterial meningitis and bacteremia. Although the virulence of this pathogen is in large part determined by its capsular polysaccharide, it produces several additional virulence factors involved in the infection mechanisms. Pneumolysin, like α-toxin as an example, is a pore forming exotoxin that can trigger cell death in macrophages and other cell types. In human macrophages it permeabilizes mitochondrial membranes causing cytochrome c release [127].

***Bacillus anthracis*** is the causative agent of anthrax a well known zoonotic disease. Three different primary forms of the disease are recognized, the cutaneous, the inhalation and the gastrointestinal form. The bacterium has two major virulence factors, the poly-D-glutamate capsular filaments that plays a major role as an invasiveness factor and a toxin complex [128]. The toxin complex is a tripartite exotoxin that consists of three polypeptide subunits: the protective antigen, the edema factor and the lethal factor. The protective antigen binds to cellular receptors and mediates the entry into the cytosol of the other two factors. The lethal factor is a Zn+ metalloprotease that inhibits the MAPK pathway causing cell death, while the edema factor is an adenylate cyclase that converts ATP to cyclic AMP and promotes lethal tissue edema [128]. Both lethal factor and edema factor inhibit acquired and innate immune responses, allowing the bacteria to multiply in the host.

***Listeria monocytogenes*** is involved in a foodborne illness characterized by gastroenteritis, meningitis, encephalitis and sepsis [129]. It is a facultative endo-cellular bacterium that grows in macrophages, epithelial cells and fibroblasts [129] and can induce apoptosis in several cell types [130]. In hepatocytes, activation of apoptosis probably contributes to the resolution of infection by: (i) elimination of infected cells and reduction of host cells for bacterial replication and (ii) allowing access of immune cells to the microorganisms. On the contrary, *Listeriae* can induce cell death to reduce the number of phagocytes, lowering antigen presentation and breaking efficient adaptative immune response. The hemolysin listeriolysin O, the most widely studied virulence factor of *L. monocytogenes* promotes apoptosis in host T cells [108], through a mechanism that involves loss of mitochondrial membrane potential [108].

***Clostridium difficile*** causes nosocomial diarrhoea in adults by colonizing the lower intestinal tract [131]. Two virulence factors, enterotoxins A and B, largely responsible for pathogenicity, are both inducers of apoptosis. In intestinal epithelial cells, the toxins produce a loss of mitochondrial membrane potential followed by release of cytochrome C [130]. The decrease of Rho protein activity appears necessary to trigger apoptosis [132]. These toxins can also trigger the extrinsic pathway [130].

### Viral infections

Viruses can only replicate inside host cells that in turn possess several defense mechanisms to limit viral infection, including cell-mediated immune response, inflammation and programmed cell death. Viruses have therefore developed several strategies to inhibit or delay cell death. On the other hand, some viruses induce apoptosis to facilitate the viral spreading and/or to kill uninfected cells of the immune system.

A large number of reports and reviews have been published on this subject that will provide valuable insights [133, 134], here we shortly report some of the newest findings on the topic.

***Herpesviridae*** family comprises double-stranded DNA viruses that cause common infections in humans. *Herpesviruses* may live latently in specific cell types for years and then be activated and cause disease. Varicella-Zoster virus (VZV) has a short replication cycle and it is the etiological agent of chickenpox and after a long latency period in ganglia along the entire neuraxis, viruses can reactivate and produce shingles and other neurological disorders. Despite the bulk of literature on the subject the molecular mechanism of VZV-induced cell death is still elusive. A recent study in a melanoma cell model suggests a potential role for Bcl-2 [135]. The authors show that Bcl-2 mRNA and protein levels decrease significantly during progression of the infection, resulting in the release of cytochrome C.

*Cytomegalovirus* has a characteristically long replication cycle and causes a sub-clinical infection in immune-competent hosts. *Cytomegalovirus* ensures its own survival in the host cells, producing a wide range of cell death suppressors [136], among these is a viral mitochondrion-localized inhibitor of apoptosis (vMIA) with a wide anti-apoptotic activity against both extrinsic and intrinsic apoptosis-inducing stimuli. It is functionally and structurally similar to Bcl-x_L_ [137] and prevents the release of pro-apoptotic factors from mitochondria by interaction with the growth arrest and DNA damage 45α (GADD45α) protein and Bcl-x_L_ [138]. Additionally, vMIA blocks Bax by binding and sequestering it at the mitochondrion [139]. *Cytomegaloviruses* also encode an inhibitor of apoptosis that suppresses caspase 8 activation. This viral inhibitor is highly conserved among mammalian betaher-pesviruses [136]. Finally Human cytomegalovirus infection is known to cause ER stress [140], that would normally result in the unfolded protein response (UPR) and apoptosis, however cytomegaloviruses encode UL38 protein that inhibits ER stress dependent apoptosis [140].

***Hepatitis B*** virus (HBV) is a small hepatotropic virus with a partially double-stranded circular DNA molecule. HBV can induce several liver diseases including asymptomatic infections, acute or fulminant hepatitis, chronic hepatitis with progression to cirrhosis and hepatocellular carcinoma. Apoptosis can play an important role in the progression of HBV infection. To date, four HBV proteins can triggers apoptosis in various processes: the large surface protein, a truncated form of the middle surface protein, the Hbx protein and HBSP [141].

HBV codes for three forms of the surface (envelope) protein, known as large, middle and small surface proteins [142]. HBV large surface protein induces apoptosis in cultured hepatoma cells by activating the ER stress pathway [142]. The protein accumulates within the ER-Golgi intermediate compartment (ERGIC) giving rise to membrane-bound vescicles that cause marked vacuolization of the cytoplasm and subsequently ER stress [142].

The HBV surface protein MHBs(t) (C-terminally truncated middle hepatitis B surface protein) is a potent regulator of TRAIL-induced apoptosis through a mechanism that requires activation of ERK2 and increased cleavage of caspases 3 and 9 [143].

*Hepatitis B* X protein (HBx) is a small regulatory protein involved in the establishment and/or maintenance of the chronic state of the infection that can induce apoptosis in several ways [144]. It inhibits c-FLIP, an inhibitor of the intrinsic apoptotic pathway, resulting in hyper-activation of caspases 8 and 3 by death signals [144]. However a more recent study demonstrates that HBx can be either pro- or anti-apoptotic in rat hepatocytes [145], inhibiting apoptosis by activating NF-κB but stimulating it if NF-κB activity is inhibited [145].

HBSP is a splice variant of the HBV DNA polymerase with a conserved BH3 domain in the N-terminus. It is expressed during viral replication and can induce apoptosis functioning as a BH3 only protein [146].

***Hepatitis C virus*** (HCV) is a small positive single-strand RNA virus of the *Flaviviridae* family. HCV infection is associated with severe liver disease that frequently evolves into chronic disease, cirrhosis, and hepatocellular carcinoma. During chronic infection it was observed an enhanced hepatocyte apoptosis and up-regulation of the death inducing ligands CD95/Fas, TRAIL and TNFα [147]. However, despite the extensive literature the role of apoptosis in chronic infection remains a matter of debate, and it also questionable if the virus increases or reduces apoptosis of infected cells. Moreover the absence of an appropriate in vitro infection model has rendered the study of this subject quite difficult and the interpretation of the data not always univocal. [147].

***Influenza A virus*** are small viruses of the family *Orthomyxoviridae* with a segmented negative-stranded RNA genome, that affects humans and animals. Influenza A virus regulates apoptosis in several ways through multiple viral proteins with both pro- and anti-apoptotic activity [148]. The flu virus can induce death through activation of TGF-β, converting it from its latent form through the viral neuraminidase activity [148]. It has also been demonstrated that overexpression of the anti-apoptotic protein Bcl-2 results in impaired virus production correlating with misglycosylation of the viral surface protein haemoagglutinin [148]. The block of apoptosis seems to be crucial for viral replication, whereas the induction of cell death may be implicated in evasion of the immune system.

***Human immunodeficiency virus*** (HIV) type 1 and type 2 are the causative agents of AIDS. The majority of studies have concentrated on the more aggressive HIV-1. HIV infection is primarily associated with a progressive decline in CD4^+^ T lymphocytes number, consequent immunodeficiency and increased susceptibility to opportunistic infections and malignancies. The main mechanism for CD4^+^ T cell depletion is enhanced apoptosis, which can be induced by HIV through multiple pathways. HIV triggers apoptosis in both infected and uninfected CD4^+^ T cells, and indeed death of uninfected cells seems to be predominant [149, 150].

HIV enters the cells through the binding of the envelope glycoprotein gp120 to the CD4 membrane receptors, along with the chemokine co-receptor CXCR4 that facilitates membrane fusion between cells to form giant multinucleated cells (syncytia) that correlate with increased death. Furthermore, death also occurs by enhanced membrane permeability due to continuous budding of virions and Viral Protein U (Vpu). Once inside the host cell, HIV proteases specifically cleave and inactivate Bcl-2 and it directly activate procaspase 8 by proteolysis [151]. Finally, other HIV proteins such as negative regulator factor (Nef), envelope glycoprotein (Env) and trans-activator of transcription (Tat) are able to trigger apoptosis in T-cells by a mechanism that involves the Fas-FasL signaling pathway.

As mentioned above HIV also kills uninfected cells through several mechanisms, that require the release of viral proteins such as gp120, Tat and Nef from infected cells into the extracellular environment. These proteins in turn trigger apoptosis in bystander cells by different mechanisms. Soluble and membrane bound gp120 binds different receptors (CD4, CXCR4 and CCR5) inducing death both by upregulating Fas and decreasing FLIP and activating the intrinsic pathway through down-regulation of Bcl-2 and up-regulation of Bax. Tat is endocytosed by neighbouring cells and up-regulates caspase 8 and FasL resulting in death of T helpers and neurons, while it appears to kill bystander T cells through up-regulation of TRAIL. Induction of apoptosis by Nef occurs through unknown mechanisms, however genes involved both in the intrinsic and extrinsic pathways have been shown to be regulated by this protein, in primary human brain microvascular endothelial cells suggesting that it can potentially induce death trough different mechanisms.

### Autoimmune diseases

A common feature of autoimmune diseases is altered tolerance to self antigens and generation of autoantibodies. Immune homeostasis and maintenance of immune tolerance are strongly dependent on apoptosis, moreover defective clearance of dying cells results in persistence of autoantigens, therefore autoimmune diseases can arise both from defective clearance of autoreactive cells or by delayed elimination of autoantigens. In addition increased apoptosis as a consequence of viral infections, gamma irradiation or other stressing conditions may contribute to disease onset. More recently it has been suggested that upon apoptosis and/or secondary necrosis autoantigens are cleaved and modified exposing novel epitopes that are recognized by the immune system, again altered or delayed clearance as well as prolonged exposure to apoptotic inducing stimuli would result in autoimmune response. Moreover formation of immune complexes would result in secretion of pro-inflammatory cytokines such as IL-8, Il-1β, TNFβ and IFN-α resulting in chronic inflammation and organ damage.

A large number of evidence support the idea that defective apoptosis of immune cells leads to autoimmune disease. Lpr and gld mice defective for the Fas signaling pathway develop lymphoadenopathy and splenomegaly and produce a large number of autoantibodies developing a disease that resembles human systemic lupus erythematosus (SLE), clearly demonstrating an essential role for the extrinsic apoptotic pathway in controlling autoreactive T and B cells and the fact that alteration in apoptosis can strongly contribute to autoimmune diseases pathogenesis [152, 153]. In humans defects of the Fas signaling pathway lead to the autoimmune lymphoproliferative syndrome (ALPS) characterized by non-malignant lymphoproliferation and autoimmunity and have increased incidence of malignancies. 70% of these patients carry germ-line heterozygous FAS mutations, while the rest have somatic FAS mutations or mutations of FAS ligand, caspase 10 and caspase 8. In most cases mutations function as dominant negatives inhibiting also the function of the wild type protein [154].

While defects in the extrinsic pathway seem to play a major role in the immune system it is becoming clear that also the intrinsic pathway participates and its alterations can contribute to autoimmune disease pathogenesis [155]. As an example Bim KO mice have been shown to accumulate lymphoid and myeloid cells and develop an autoimmune disease, [156, 157]. While no mutations of the BH3 only proteins have been described in patients with autoimmune diseases, reduced levels of Bim were reported in a patient with ALPS and over-expression of pro-survival members of the bcl2 family have been reported in SLE [155].

As mentioned above altered clearance of apoptotic cells also contributes to autoimmune diseases pathogenesis. In fact MGF-8 (a protein essential for macrophage clearance of apoptotic cells) defective mice also produce a large number of autoantibodies and develop a SLE-type autoimmune disease [152]. This is probably due to release of cellular material from apoptotic cells that have not been cleared by phagocytosis and undergo secondary necrosis. These cellular antigens would activate autoimmunity. Along the same line mice defective for the complement component C1q develop a SLE-like glomerulonephritis. Moreover defective clearance of apoptotic cells has been demonstrated in patients with SLE [158] and macrophages from at least 50% of these patients show reduced phagocytosis [159].

### Concluding remarks

Over 50 years of research in the field of cell death have clarified many aspects of this fundamental process and brought to the attention of scientist its role in a large number of different diseases, however exploitation of this knowledge in therapy is only at its early steps. We expect that in the following years more approaches based on control of the different forms of cell death will enter the clinical practice. Many problems need to be solved, of course, such as activation of alternative death pathways when one is pharmaceutically blocked, in diseases where survival of the target cell is the final goal, as well as unwanted death of “innocent bystanders”, when attempting to kill pathological cells such as in anti-tumor therapies.
